# Light and Temperature Synchronizes Locomotor Activity in the Linden Bug, *Pyrrhocoris apterus*

**DOI:** 10.3389/fphys.2020.00242

**Published:** 2020-04-02

**Authors:** Magdalena Maria Kaniewska, Hana Vaněčková, David Doležel, Joanna Kotwica-Rolinska

**Affiliations:** ^1^Institute of Entomology, Biology Centre of Academy of Sciences of the Czech Republic, České Budějovice, Czechia; ^2^Faculty of Science, University of South Bohemia, České Budějovice, Czechia

**Keywords:** circadian clock, *Pyrrhocoris apterus*, thermoperiod, photoperiod, synchronization, entrainment, constant light, temperature compensation

## Abstract

Circadian clocks are synchronized with the external environment by light and temperature. The effect of these cues on behavior is well-characterized in *Drosophila*, however, little is known about synchronization in non-model insect species. Therefore, we explored entrainment of locomotor activity by light and temperature in the linden bug *Pyrrhocoris apterus* (Heteroptera), an insect species with a strong seasonal response (reproductive diapause), which is triggered by both photoperiod and thermoperiod. Our results show that either light or temperature cycles are strong factors entraining *P. apterus* locomotor activity. *Pyrrhocoris* is able to be partially synchronized by cycles with temperature amplitude as small as 3°C and more than 50% of bugs is synchronized by 5°C steps. If conflicting zeitgebers are provided, light is the stronger signal. Linden bugs lack light-sensitive (*Drosophila*-like) cryptochrome. Notably, a high percentage of bugs is rhythmic even in constant light (LL) at intensity ∼400 lux, a condition which induces 100% arrhythmicity in *Drosophila*. However, the rhythmicity of bugs is still reduced in LL conditions, whereas rhythmicity remains unaffected in constant dark (DD). Interestingly, a similar phenomenon is observed after temperature cycles entrainment. Bugs released to constant thermophase and DD display weak rhythmicity, whereas strong rhythmicity is observed in bugs released to constant cryophase and DD. Our study describes the daily and circadian behavior of the linden bug as a response to photoperiodic and thermoperiodic entraining cues. Although the molecular mechanism of the circadian clock entrainment in the linden bug is virtually unknown, our study contributes to the knowledge of the insect circadian clock features beyond *Drosophila* research.

## Introduction

Majority of organisms experience periodic changes in the environment, such as daily alternations of light, dark and temperature. Circadian clocks are time-measuring mechanisms that evolved as an adaptation to prepare for these external changes and thus to anticipate events like sun dawn, the flowering of particular plants, or avoiding predators. Circadian clocks free run under constant conditions, such as constant darkness (DD), with periodicity close to 24 h. This free-running period (tau, τ) remains almost constant over a physiologically relevant range of temperatures, a phenomenon termed “temperature compensation” (Dunlap et al., 2004). Some clock mutants show impaired temperature compensation, whereas other mutations are temperature-independent (Hamblen et al., 1998; Matsumoto et al., 1999; Rothenfluh et al., 2000; Singh et al., 2019).

Circadian clocks can (and need to) be synchronized with the environment. The strongest cues, the “zeitgebers” (from German Zeit: time, Geber: giver), are light-dark cycles (LD), and temperature cycles (TCs), but circadian clocks can also be synchronized by other cues including food availability and social interaction (Levine et al., 2002; Dunlap et al., 2004; Sharma and Chandrashekaran, 2005; Shaw et al., 2019).

The circadian clock of the fruit fly, *Drosophila melanogaster*, the premiere insect model, is very well understood at the genetic, molecular and anatomical levels. The clock consists of several interlocked transcriptional-translational feedback loops (reviewed in Hardin, 2011; Ozkaya and Rosato, 2012; Tataroglu and Emery, 2015). The light input is mostly mediated by protein CRYPTOCHROME (CRY), a deep brain photoreceptor expressed in clock neurons (Emery et al., 2000). However, flies with mutated or completely removed *cry* gene can be still synchronized by LD, although the synchronization requires more days (Stanewsky et al., 1998; Dolezelova et al., 2007). This second synchronization pathway involves opsins in the compound eyes, ocelli, and the Hofbauer-Buchner eyelet (for review on the light input see Helfrich-Förster, 2019).

In contrast to light entrainment, the mechanism underlying temperature synchronization is less understood. Moreover, temperature affects circadian timekeeping in different ways. While the τ is temperature compensated, the actual daily activity profile and phase are temperature-dependent. Periodic TCs can synchronize the clock, whereas temperature pulses and steps can reset its phase (Sharma and Chandrashekaran, 2005; Tomioka and Yoshii, 2006).

The daily activity profile reflects the life strategy of a specific species used for coping with the environment. Adults of *D. melanogaster* display clear bi-modal locomotor activity with mid-day siesta, although the behavioral pattern is more complex under natural conditions (Menegazzi et al., 2012; Vanin et al., 2012). At simplified regimes in a laboratory with constant temperature and alternation of LD without any ramp of light intensity and color, flies display clear morning and evening activity peaks. At 25°C and LD 12:12 the morning peak corresponds to light-on signal and the evening peak matches light-off. At 18°C, the morning peak is delayed and evening peak advances, whereas at 29°C the morning peak starts already during DD and the evening peak is delayed to DD (Majercak et al., 1999). A similar trend of centering activity to mid-day at low temperatures and spreading activity to morning and evening at high temperatures was reported for the house fly, *Musca domestica*, although the actual molecular mechanism behind the trend differs between *D. melanogaster* and *M. domestica* (Bazalova and Dolezel, 2017).

*Drosophila* behavioral rhythm can be entrained by TCs even in constant light (LL) and requires functional *norpA* and *nocte* genes (Glaser and Stanewsky, 2005). When conflicting TCs and LDs were applied, the light seems to be the dominant signal (Yoshii et al., 2010). However, detailed systematic comparison of conflicting zeitgebers revealed that the light dominates temperature for maximal misalignments, but smaller delays of LD relative to TCs lead to rhythms that predominantly follow the temperature cue. Notably, certain alignments of TCs and LD result in dramatic behavioral disruption during and even after exposure to sensory conflicts (Harper et al., 2016).

Although the circadian clock toolkit is remarkably conserved between the *D. melanogaster* and mammals, certain important differences exist. For instance, mammals lack photosensitive *Drosophila*-like CRY responsible for the light input, instead, two closely related non-photosensitive CRYs work as transcriptional repressors in the feedback loop (Kume et al., 1999). Interestingly, close inspection of insect circadian clocks revealed various combinations of mammalian-like CRY and *Drosophila*-like CRY in different insect species (Yuan et al., 2007; Tomioka and Matsumoto, 2010). In the present study, we explored the effect of the light and temperature cycles on the entrainment of the locomotor activity of the linden bug, *Pyrrhocoris apterus* (Heteroptera), insect species that lacks light-sensitive *Drosophila*-like CRY and instead contains mammalian-like CRY (Bajgar et al., 2013a, b).

The linden bug has been used to elucidate eco-physiological aspects of insect seasonality (Dolezel et al., 2007; Kostal et al., 2008; Ditrich et al., 2018), particularly the photoperiodically induced reproductive diapause (Saunders, 1983, 1987) and the hormonal regulation of this reproductive arrest (Hodkova, 1976; Smykal et al., 2014; Urbanova et al., 2016). The diapause is induced by short photoperiods during the last larval stages resulting in non-reproductive adults. Although *P. apterus* clearly distinguishes between long and short photoperiods at ambient temperature such as 25°C, the photoperiodic response curve is affected by temperature and shifts to longer photoperiods at low temperatures and to short photoperiods at high temperatures, respectively (Numata et al., 1993). Diapausing *P. apterus* females are characterized by reduced locomotor activity, with the activity peak advanced when compared to the activity of reproductive females (Hodkova et al., 2003). Comparable phase advance is observed in the locomotor activity of nymphs (Kotwica-Rolinska et al., 2017). Apart from this basic characterization of locomotor activity in reproductive and diapause animals, no information is available on the role of light and temperature in *P. apterus* circadian entrainment. Therefore, the goal of this study was to provide basic characterization of the linden bug locomotor activity under various thermoperiodic and photoperiodic regimes and to describe the effect of different zeitgebers on the circadian clock entrainment.

## Materials and Methods

### Insect Rearing and Locomotor Activity Measurements

The colony of *P. apterus* from the Czech line Oldrichovec (Pivarciova et al., 2016) was maintained in the laboratory under diapause-preventing conditions (photoperiod LD 18:6 and constant temperature 25°C). Males 3–5 days after adult ecdysis were individually transferred to the LAM (Large Activity Monitors, Trikinetics, Inc., Waltham, MA, United States), supplemented with food and water *ad libitum* and activity was recorded every 5 min during the entire experiments. Unless specified, bugs were entrained for 5 days to a new photo- and thermoregime. Afterward, bugs were released for 12 days into constant conditions (constant temperature with either constant darkness or constant light) to determine their τ. For assessing entrainment ability of *P. apterus* to temperature cycles, males were kept in specific entrainment conditions for 10 days. The actual temperature profile was recorded during the entire experiment by Drosophila Environmental Monitors (Trikinetics, Inc., Waltham, MA, United States). All activity measurements were performed in the Cooled Incubator Sanyo MIR-154 equipped with a built-in electronic timer. Temperature steps between cryophase and thermophase were completed within approximately 20 min and temperature fluctuations during experiments did not exceed ±0.5°C. Light intensity (∼white light, see Supplementary Figure 1 for the spectrum) in LD and LL experiments was ∼400 lx.

### Locomotor Activity Analysis

Lomb-Scargle periodogram in ActogramJ (Schmid et al., 2011) was used to determine the rhythmicity of bugs and τ in constant conditions, and double-plotted actograms were further inspected by eye (Refinetti et al., 2007; Brown et al., 2019). Three categories were defined: (1) *Strongly rhythmic* males: periodogram peak crossed the significance line and PN value calculated by ActogramJ software was >65. (2) *Weakly rhythmic* males: periodogram peak crossed the significance line and PN values were within 35–65. (3) *Arrhythmic* males: periodogram peak either did not cross the significance line or periodogram peak crossed the significance line but PN value was <35 (Pivarciova et al., 2016).

Daily profile of locomotor activity was analyzed in ActogramJ software (Schmid et al., 2011). The activity of all rhythmic individuals in a particular group was averaged, smoothed (Gaussian smooth 3) and displayed as double-plotted actogram. The activity of the last entrainment day and first 2 days in constant conditions were plotted in 5 min resolution for average from all animals in the experiment without any smoothing. For comparison of the activity phase at 21.5°C, the activity of all measured individuals was averaged to 1-h bins and plotted without any smoothing.

Ability to synchronize locomotor activity to TCs was assessed from 10-day recordings under constant dark and long thermoperiod consisting of 18 h of thermophase and 6 h of cryophase differing by 1, 3, 5, or 7°C (18–19, 18–21, 18–23, 18–25, 24–25, 22–25, and 20–25°C). Periodic locomotor activity was determined by the Chi-square periodogram algorithm in ActogramJ (Schmid et al., 2011). If analyses showed significant rhythm of 24 h (±15 min) and actograms passed visual confirmation, males were considered as “synchronized.” The reference locomotor activity under constant dark at 18 or 25°C was analyzed analogously with Chi-square periodogram.

### Statistical Analysis

The differences between τ were tested for statistical significance by *t*-test and Kruskal–Wallis test with Dunn’s *post hoc* test using Graphpad7 software (Prism, La Jolla, CA, United States).

## Results

### *Pyrrhocoris apterus* Locomotor Activity Is Synchronized by Light and Temperature Cycles

The first experiments addressed if the locomotor activity of *P. apterus* can be synchronized by light or temperature. Since the Oldrichovec strain has τ longer than 24 h (Pivarciova et al., 2016), simple alignment of activity to 24 h zeitgebers reliably indicates the successful synchronization. Therefore, to simplify the assay, males developing at 25°C and LD 18:6 were transferred to a new regime where ZT0 corresponded to ZT0 during the development.

**FIGURE 1:**
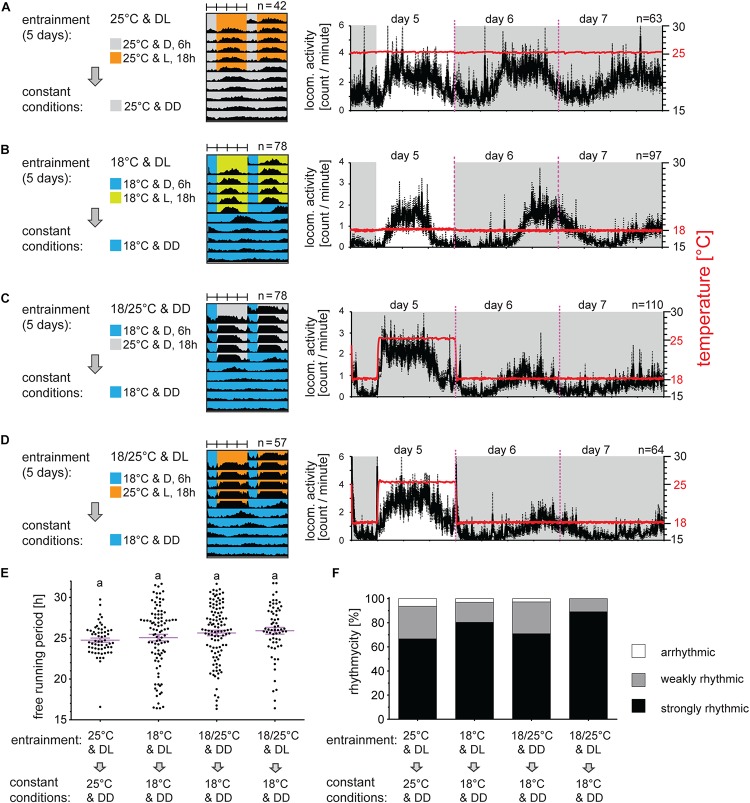
Long periods of light and/or temperature synchronize locomotor activity in the linden bug, *Pyrrhocoris apterus*. Adult males raised under long photoperiod D6:L18 at 25°C were entrained for 5 days either by long photoperiods **(A,B)**, long thermoperiods **(C)**, or combination of both light and temperature **(D)** and released to constant dark at either 25°C **(A)** or 18°C **(B–D**). Double-plotted actograms represent the average activity of all males rhythmic under DD conditions [see panel **(F)** for the rhythmicity]. Detailed activity profile is plotted from all males for day 5–7 [right panels in **(A–D)**]. The actual temperature recorded during the experiment is shown in red (right *y*-axis). The rhythmicity under constant conditions was determined from 10 days and is presented as the free-running period of each individual male (dots) and mean ± SEM shown in magenta **(E)**, there is no difference in the free-running period between groups (*p* > 0.05, Kruskal–Wallis). The same small letters above categories indicate no statistical difference. The percent of arrhythmic, weakly rhythmic and strongly rhythmic males is shown in panel **(F)**.

Data from individual bugs are very noisy. Until now we were not able to reliably determine the precise time of the locomotor activity onset, off-set or acrophase for a single bug, which is usually used for determining the exact phase of activity (Refinetti et al., 2007; Brown et al., 2019). Therefore, the average activity of all bugs was analyzed. Males exposed to LD 18:6 and constant 25°C are active during the majority of the photophase with a small delay of activity onset and anticipation of the light off (Figure 1A). The same photoperiod at a lower temperature (18°C) results in a narrower peak with the activity onset comparable to onset at 25°C, but the activity offset was 6 h before light off at 18°C (Figure 1B). When released to DD, the first activity cycle is clearly delayed at 18°C (compare Figures 1A,B) and the locomotion then continues rhythmically (Figure 1B). Locomotor activity is also clearly synchronized by TCs in DD (Figure 1C). Notably, the activity onset followed the temperature rise with only minimal delay and this early rise of activity is not observed in DD at a constant temperature. This startling behavior represents most probably a direct response to the temperature rise (masking effect). Although a majority of males were strongly rhythmic in DD, the τ values were quite dispersed after TC entrainment (Figure 1E), which resulted in a noisy average activity and thus the offset is visible only for the first two DD cycles. A synergistic combination of the photoperiodic and thermoperiodic entrainment (Figure 1D) synchronized locomotion with the onset during the photophase-thermophase comparable to the timing of the onset observed under LD at 25°C (Figure 1A). Interestingly, the startling effect to the temperature rise is absent in these conditions. The activity after all entrainment regimes continues rhythmically in DD with average τ longer than 24 h (Figure 1E and Supplementary Table 1) and shows no significant difference of the τ between bugs synchronized by different entraining protocols (*p* > 0.05 Kruskal–Wallis test). The τ values are quite dispersed in DD conditions at 18°C (Figure 1E) and extremely short (below 18 h) and long τ (above 30 h) are observed. The correlation analysis between significance level of the Lomb-Scargle periodogram (PN value) and τ (Supplementary Figure 2) shows that the shortened τ correlate weakly with the lower PN values (Spearman correlation *p* < 0.001, *r* = −0.355). We cannot rule out that the extremely short τ could be a result of false-positive signal recognition from noise by the Lomb-Scargle periodogram algorithm (Zielinski et al., 2014). On the other hand, extremely long periods (τ > 30 h) do not show reduced power (PN) values (Spearman correlation *p* > 0.05, *r* = 0.0823) and eye-inspection further confirmed the presence of the long τ. Majority of bugs shows strong rhythmicity in DD and constant temperature after all entrainment regimes, with only a small portion of bugs being arrhythmic (Figure 1F). Combination of the photoperiodic and thermoperiodic entrainment produced the highest percentage of strongly rhythmic bugs in DD with no arrhythmic individuals (Figure 1F).

### Light Is a Stronger Signal Than Temperature

**FIGURE 2:**
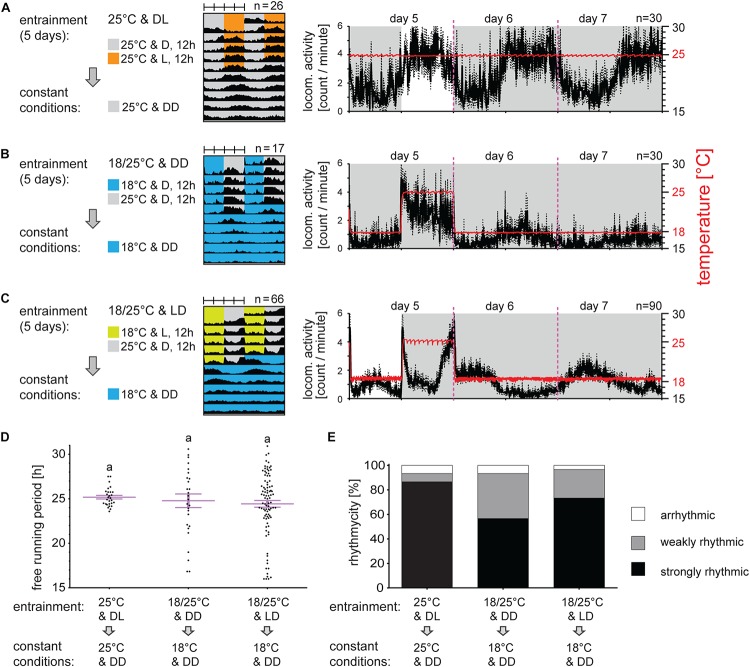
Light is a stronger signal than the temperature for locomotor activity synchronization in *P. apterus*. Adult males developing under long photoperiod (D6:L18) were entrained by 5 days of short photoperiod D12:L12 **(A)**, short thermoperiod **(B)** or conflicting cycles of short photoperiod and thermoperiod where the photophase was combined with the cryophase and the thermophase was combined with the scotophase. After 5 day synchronization, males were released to DD at either 25°C **(A)** or 18°C **(B,C)**. Double-plotted actograms represent the average activity of all males rhythmic under DD conditions [see panel **(E)** for the rhythmicity]. Detailed activity profile is plotted from all males for day 5–7 (right panels in **(A–C)**). The actual temperature recorded during the experiment is shown in red (right *y*-axis). The rhythmicity under constant conditions was determined from 10 days and is presented as the free-running period of each individual male (dots) and mean ± SEM shown in magenta **(D)**, there is no difference in the free-running period between groups (*p* > 0.05, Kruskal–Wallis test). The same small letters above categories indicate no statistical difference. The percent of arrhythmic, weakly rhythmic and strongly rhythmic males is shown in panel **(E)**.

In the second set of experiments, males were entrained by short (12:12) photoperiodic and/or TCs. Males transferred from LD 18:6 to LD 12:12 and constant 25°C needed approximately two to three cycles for synchronization. The activity in LD 12:12 then covered the entire photophase starting during the scotophase and the activity continued in phase during DD (Figure 2A). Under short TC, locomotor activity raised immediately with temperature (Figure 2B). Conflicting LD and TC regime (photophase combined with cryophase, and scotophase matching thermophase) resulted in a very mild peak during the photophase/cryophase and clearly bimodal activity during the beginning and end of scotophase/thermophase (Figure 2C). Release into constant conditions (DD and 18°C) clearly indicates that the activity phase corresponded to the photophase, thus the activity during the thermophase was clearly a masking effect (compare Figures 2A–C). Different cycling condition, even conflicting LD and TC regime did not affect bugs rhythmicity nor the τ of their locomotor activity upon release into DD (Figures 2D,E).

### Thermophase Defines the Phase of Activity Under TC

**FIGURE 3:**
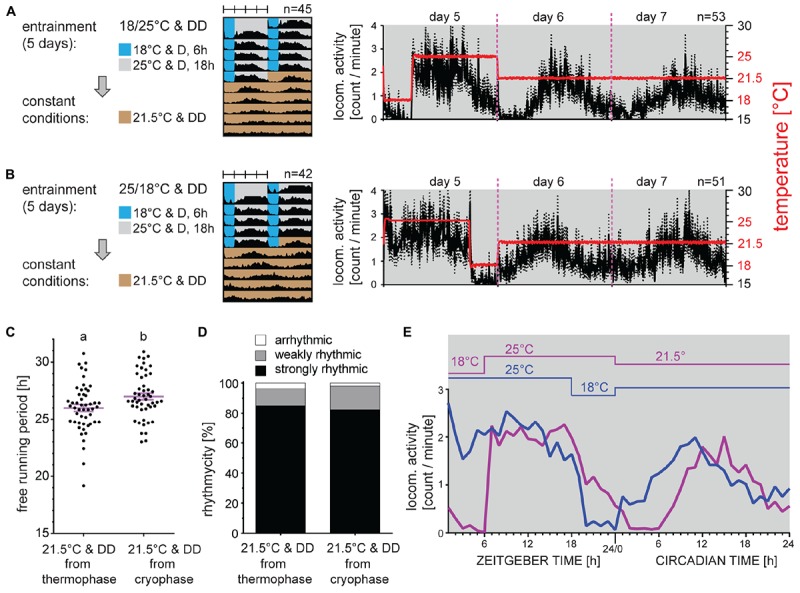
Phase of activity is defined by the thermophase. Adult males developing under long photoperiod (D6:L18) were entrained by 5 days of constant dark and long thermoperiod 18:6 with thermophase at 25°C and cryophase at 18°C. Then, males were released to intermediate temperature (21.5°C) either from the thermophase **(A)** or from the cryophase **(B)**. Double-plotted actograms represent the average activity of all males rhythmic under constant conditions (see panel **(D)** for the percent rhythmicity). Detailed activity profile is plotted from all males for day 5–7 [right panels in **(A,B)**]. The actual temperature recorded during the experiment is shown in red (right *y*-axis). Detail activity profiles averaged into 1-h bin are shown for the last day of thermoperiodic entrainment and the first day of constant conditions **(E)**. The rhythmicity under constant conditions was determined from 10 days and is presented as the free-running period of each individual male (dots) and mean ± SEM shown in magenta **(C)**, where different small letter above categories indicate statistical difference *p* < 0.05 (*t*-test). The percent of arrhythmic, weakly rhythmic and strongly rhythmic males is shown in panel **(D)**.

To further characterize thermoperiodic entrainment, males were synchronized by long TC consisting of 18 h at 25°C and 6 h at 18°C. Consistently with TC-induced behavior described in Figure 1C, the activity raised immediately with temperature (Figures 3A,B). To determine if the phase of activity is influenced by the thermophase or cryophase, males were released to intermediate 21.5°C either from 25°C, or from 18°C (Figures 3A,B). Clearly, the activity phase corresponded to previous thermophase (Figures 3E) and no phase change is observed in either of tested conditions, when compared to activity during the entrainment. Although the percent rhythmicity in DD was comparable in both groups, a small significant increase in τ was observed for males released from the thermophase (Figures 3C,D).

### Synchronization by TC

**FIGURE 4:**
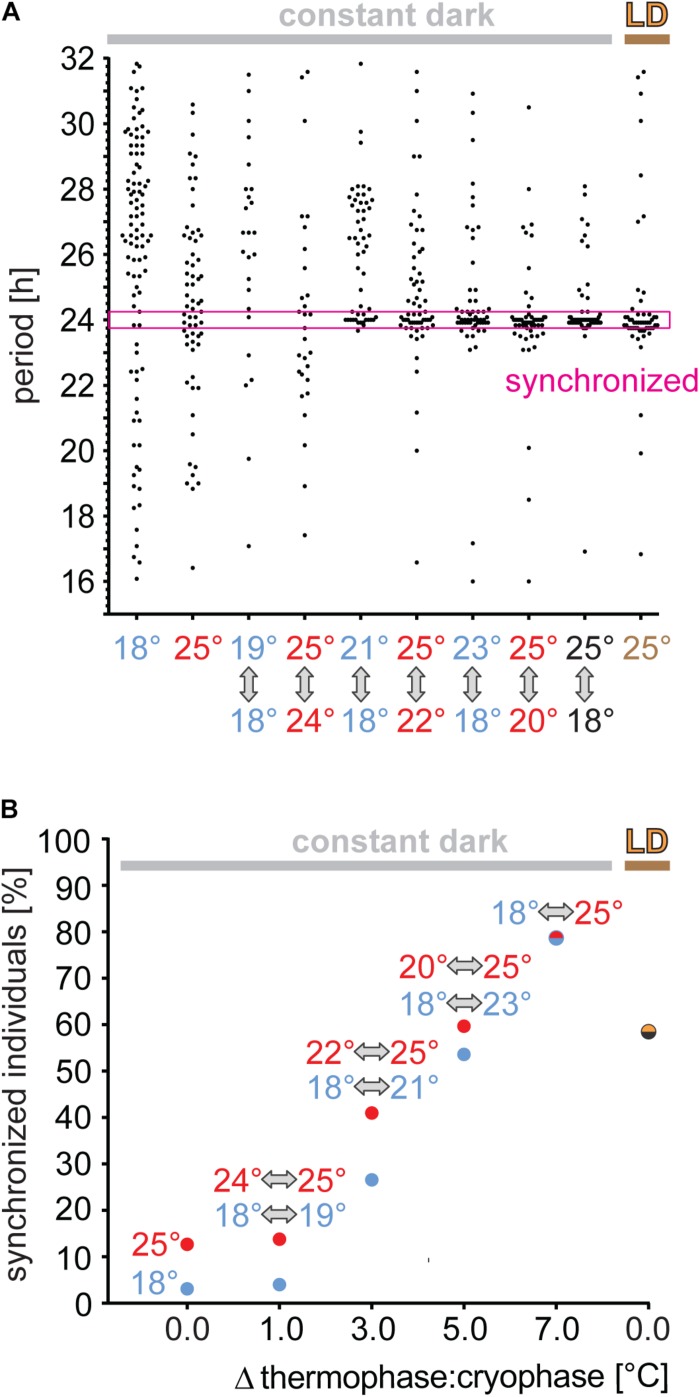
Synchronization efficiency to thermoperiodic cycles depends on the temperature range. Adult males developing in long photoperiod (L18:D6) were released to thermoperiodic cycles (thermophase 18 h matched the previous photophase of LD) with 1, 3, 5, or 7°C steps. Periodicity of locomotor activity is determined from 10 days with chi-square algorithm and males with period 24 h (±15 min) are considered as synchronized [magenta window in panel **(A)**]. As a positive control, males synchronized by LD regime at a constant 25°C (brown) are used. As a reference, τ in constant DD at 18°C (blue) or at 25°C (red), respectively, are plotted. The percentage of synchronized males is shown for each temperature steps in panel **(B)**.

To determine the sensitivity of *P. apterus* to TC, males were released from LD to TC in the constant dark, where thermophase corresponded to the previous photophase. Since the majority of males have τ that differs from 24 h by more than 15 min (the very left data in Figures 4A,B), males with periodic activity within 24 h (±15 min) were considered as “synchronized.” Clearly, 1°C range of TC is not sufficient to synchronize locomotion, whereas 3°C synchronized either ∼one quarter (18/21°C) or ∼40% (22/25°C) of males. The percent of synchronized males was higher with a higher difference between thermo- and cryophase, reaching up to ∼80% for the 7°C difference. Thus, temperature cycles of relatively high amplitude were even more potent for synchronizing bugs activity than LD cycles (intensity of light ∼400 lux), which synchronized only ∼60% of males.

### Locomotor Activity Can Be Synchronized by TC in Constant Light

**FIGURE 5:**
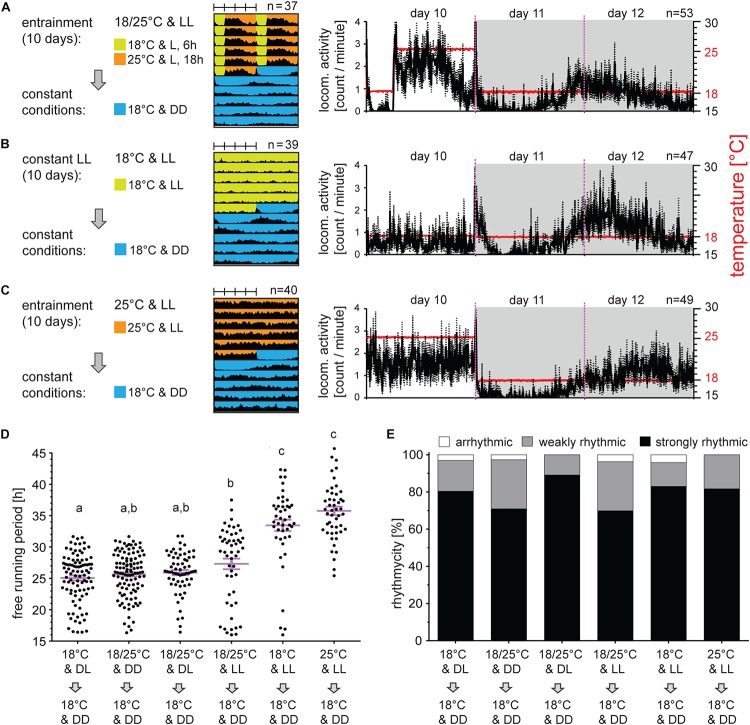
Locomotor activity can be synchronized by temperature cycles in constant light. Adult males developed under long photoperiod (D6:L18) and were exposed either to 10 days of constant light and 18°C or 25°C [panels **(B**,**C)**, respectively] or to constant light and long thermoperiod **(A)**. Afterward, males were released to constant DD at 18°C. Double-plotted actograms represent the average activity of all males rhythmic under DD conditions [see panel **(D)** for the rhythmicity]. Detailed activity profile is plotted from all males for day 10–12 [right panels in **(A–C)**]. The actual temperature recorded during the experiment is shown in red (right *y*-axis). The rhythmicity under constant conditions was determined from 10 days and is presented as the free-running period of each individual male (dots) and mean ± SEM shown in magenta **(D)**, where different small letter above categories indicate statistical difference *p* < 0.05 (Kruskal–Wallis test with Dunn’s *post hoc* test). The percent of arrhythmic, weakly rhythmic and strongly rhythmic males is shown in panel **(E)**.

To further test the capacity of TC in synchronization of *P. apterus* activity, males were exposed for 10 days to LL and TC. The activity rose immediately with the temperature during the thermophase and felt down during the cryophase, mimicking temperature cycles (Figure 5A). When exposed to LL and constant temperature, males at 25°C were more active than males at 18°C (Figures 5B,C). At an individual level, a significant portion of bugs displayed clear rhythm in LL and constant temperature (for the description of this behavior see the next chapter), however, their activity was not synchronized. This eventually resulted in a clearly arrhythmic locomotor activity pattern when the activity of all bugs was averaged (Figures 5B,C).

When bugs were kept in LL with TC and then released to constant DD and 18°C, τ of all rhythmic bugs was slightly longer (27.31 h) but not statistically different from bugs entrained by light:dark cycle (25.07 h) and/or TC (25.64 h) (Figure 5D). If only strongly rhythmic bugs were compared, τ after combined LL and TC entrainment was significantly different from all other groups (Supplementary Figure 3). Bugs released from LL without TCs as a synchronizing agent, showed clear and significant extension of τ (33.45 h if released from LL at 18°C and 35.76 h if released from LL at 25°C) (Figure 5D). In all cases, bugs released from LL were only rarely arrhythmic, and the majority of bugs were strongly rhythmic (Figure 5E).

### Rhythmicity Is Impaired in Constant Light or Constant Thermophase Conditions

Given the relatively solid rhythmicity observed in LL, we sought to further explore activity resembling day versus night conditions. Males entrained to LD at 25°C and released to DD at 25°C were arrhythmic in 6.35% and weakly rhythmic in 26.98%. Release to LL from the same entrainment regime produces arrhythmicity in 17.86% and weak rhythm in 41.07%.

**FIGURE 6:**
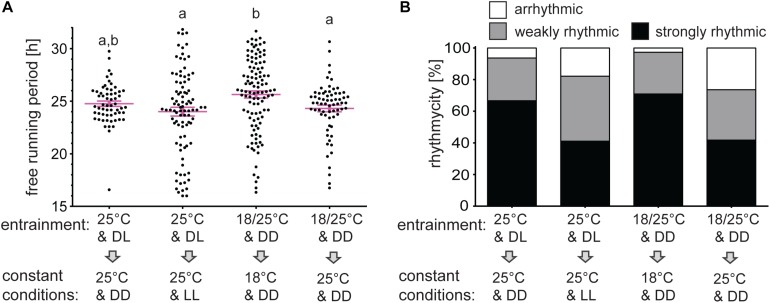
Rhythmicity in constant “day” conditions is lower than in constant “night” conditions. Adult males were entrained for 5 days in long photoperiod regime D6:L18 at 25°C and released to DD or LL at 25°C. The second group of males was entrained to long thermoperiod 18°C/25°C in DD and then released to constant cryophase of 18°C or constant thermophase of 25°C. The percent of rhythmic males **(B)** and the free-running period **(A)** differ between groups. The different small letter above categories indicates statistical difference *p* < 0.01 (Kruskal–Wallis test with Dunn’s *post hoc* test).

In the following experiment, males were entrained by TCs and released either to constant cryophase or to constant thermophase. The percent rhythmicity of males at cryophase (2.73% arrhythmic, 26.36% weakly rhythmic) were comparable to rhythmicity in DD at 25°C, whereas males released to thermophase showed reduced rhythmicity (26.37% arrhythmic, 31.87% weakly rhythmic), similarly to rhythmicity in LL at 25°C (Figure 6B and Supplementary Table 1).

The mean τ is significantly longer in constant cryophase than in constant thermopase after thermoperiodic entrainment (25.64 h at 18°C & DD versus 24.31 h at 25°C & DD). Similar, but the non-significant trend is observed in case of photoperiodic entrainment (24.76 h at 25°C and DD versus 24.31 h at 25°C and LL) (Figure 6A and Supplementary Table 1).

## Discussion

This study explored the role of photoperiod and thermoperiod as zeitgebers in *P. apterus* and clearly indicate that either light or temperature is sufficient to synchronize their locomotor activity. Under TC conditions, the activity of bugs followed immediately the rise of temperature, resembling masking effect. Similar earlier onset of the activity with temperature cycles when compared to LD cycles was observed also in *Gryllus bimaculatus* (Kannan et al., 2019), honey bees (Moore and Rankin, 1993) and *D. melanogaster* (Boothroyd et al., 2007). *Drosophila* kept in reverse photo- and thermoperiod is active still during the photophase (without any increase in activity during scotophase), however, the evening peak is advanced by ∼5–6 h (Currie et al., 2009). The authors concluded that this effect apparently required interaction between the light- and temperature-dependent entrainment mechanisms because it produced an increase in activity at a time of day when neither light nor temperature elicited this effect on their own. In linden bug, the masking effect was particularly strong in conflicting zeitgebers, when bugs showed bi-modal activity during thermophase. However, when released to a constant temperature, the bi-modal disappeared and the activity peak corresponded and matched the almost negligible peak from the photophase (Figure 2C). In nature, these two zeitgebers act synergistically together in order to adjust the temporal activity to environmental conditions. When temperature and light cycles are aligned, linden bugs are showing orchestrated rhythmic activity without any periods of hyperactivity, a situation caused by rapid changes in the ambient temperature in the beginning and the end of the thermophase. Additionally, when both zeitgebers participate synergistically in the entrainment, the stability of the rhythm in constant darkness increases is characterized by an increased % of rhythmic bugs. When LD and TC were applied in maximal misalignment, light dominated temperature. Similarly, the dominance of light over temperature was reported for *Drosophila* (Yoshii et al., 2010) and for the cricket *G. bimaculatus* (Komada et al., 2015; Kannan et al., 2019).

In case of the cricket, this conclusion was obtained from the fact that crickets entrain to the new photoregime much faster (5 cycles) than to the new thermoregime (17 cycles). Here it is important to note that crickets have both type of cryptochromes: the mammalian-like CRY which functions as a transcription repressor, and the *Drosophila*-like CRY that should serve as an efficient light receptor (Tokuoka et al., 2017). It would be very interesting to see if the linden bug, which lacks *Drosophila*-like CRY (Bajgar et al., 2013b), has a lower sensitivity to light. Unfortunately, the relatively low and particularly noisy locomotor activity makes analysis of phase shifts (determining activity on-set, off-set or acrophase) at an individual level virtually impossible. However, the noisy activity records do not prevent reliable determination of the free-running period for individual bugs (Refinetti et al., 2007; Zielinski et al., 2014; Brown et al., 2019). In this context, it is remarkable that ∼40% of males were strongly rhythmic and another ∼40% weakly rhythmic in LL with an intensity of 400 lux (Figure 6), whereas even much weaker light intensities cause complete arrhythmicity in *Drosophila* (Saunders, 1997).

When linden bugs were released to DD after LL, the τ was prolonged up to 33.45 h. Similar observations, called after-effect of LL, were observed in mice, cockroaches and chaffinches (Pittendrigh, 1960; Aschoff, 1984). After several weeks in DD animal’s τ shortens back to ∼24 h. The duration of our experimental set up in the linden bug did not allow us to determine the expected “return” of τ back to “normal” values observed in DD.

Thermocycles in LL can force linden bugs to align their activity to temperature changes. This involves a masking effect since insects, as ectothermic animals, are very sensitive to temperature. This masking effect was shown for temperature cycling to enforce the rhythmic activity of clock mutant flies in both LL and DD conditions (Yoshii et al., 2002). On the other hand, it was also shown in *Drosophila*, that temperature cycles in LL do not only provoke rhythmic activity but also synchronize molecular machinery of the clock (Glaser and Stanewsky, 2005; Currie et al., 2009). Are temperature cycles in LL conditions entraining the clock in the linden bugs? Different τ between bugs exposed to TC and bugs exposed to constant temperatures suggest that TC did not serve only as a synchronization cue in LL (note that at individual level linden bugs are often rhythmic in LL, but their activity is not synchronized between each other). τ of bugs synchronized by TC in LL is significantly shorter than τ of bugs from LL conditions, however, it still does not reach values observed in bugs synchronized by LD cycles or TC implemented in constant darkness (Supplementary Table 1). This observation shows that TC cycles can reduce, but not overcome the after-effect of LL signifying the notion of the stronger impact of the light on *P. apterus* circadian clock.

The rhythmicity of linden bug in different regimes can be approached from a different perspective. Although often rhythmic in LL, the deteriorating effect of the LL on the rhythmicity is clearly observed. The behavior is noisier which is revealed in our quantitative analysis as a higher percentage of weakly rhythmic individuals. The same situation occurs when TCs are used as entraining cues and then bugs are released to the constant thermophase. Because light phase and higher temperature are conditions which bugs are experiencing during the day, we hypothesize that constant “day-like” conditions are in some manner disruptive for the function of the clock. This effect is relatively mild, because rhythmicity of only some portions of animals was affected in this experimental setup. Interestingly, bugs which are still rhythmic in constant thermophase after TC entrainment maintain τ close to 24 h, which shows that the temperature compensation of the clock is not affected. To our knowledge, there is no report in the literature of the constant thermophase to be equivalent to LL conditions. Contrary, two *Drosophila* studies, which had identical experimental set up like ours, showed that releasing flies to constant thermophase after TC entrainment did not affect their rhythmicity (Boothroyd et al., 2007; Currie et al., 2009).

Alternative explanation for observed behavior could be that the high temperature step-up could affect the clock function. Somewhat related observation of the impact of the relative temperature on the functionality of the clock was described in one of *Drosophila* studies. Yoshii et al., 2007 showed that, while single temperature step-down of 10°C can evoke several cycles of behavioral rhythmicity in wild type, *per*^*S*^ and *per*^*L*^ flies kept in LL, the single temperature step-up does not have this potential and all flies lines show arrhythmic behavior (Yoshii et al., 2007).

Difference between linden bug and *Drosophila* was revealed in other sets of experiments. When flies are released from TCs to a constant intermediate temperature, the phase of activity depends, if they were released from thermophase (phase delay) or cryophase (phase advance) (Currie et al., 2009). In the case of *P. apterus* we did not observe any change of the activity phase, and bugs clearly follow phase set up by the thermophase during the entraining conditions. Those results could suggest that for linden bug temperature entrainment works in a slightly different manner than for *Drosophila*, however underpinning mechanism of entrainment still needs to be elucidated.

Different insect species display different sensitivity to temperature cycling amplitude. For example, honeybees are not able to entrain to TCs with an amplitude below 10°C (Moore and Rankin, 1993), whereas *Drosophila* is able to entrain even to temperature oscillations of 1.5–4°C (Wheeler et al., 1993; Currie et al., 2009). Thus the 3–5°C minimal amplitude needed for synchronization of *P. apterus* seems to fit the range observed in insects.

Temperature can effectively synchronize behavior and produce periodic activity patterns even in circadian clock mutants *per*^01^, *tim*^01^, and *cyc*^01^, although the one-peak-profile determined in arrhythmic mutants clearly differs from the bi-modal activity characteristic for rhythmic flies (Currie et al., 2009). Similarly, rhythmic behavior was observed in natural conditions and was further confirmed in a semi-natural laboratory environment (Vanin et al., 2012).

Over the past few years, details of the molecular mechanism of the temperature entrainment of the *Drosophila* circadian clock started to be unveiled (Miyasako et al., 2007; Wolfgang et al., 2013; Maguire and Sehgal, 2015; Roessingh et al., 2019). It is however unknown, if the mechanisms described for *Drosophila* are universal for all insects. Thus, it will be interesting to explore the phenomenon of the temperature entrainment in the other insect models, like *P. apterus*, with circadian clock repertoire slightly different than *Drosophila* (Bajgar et al., 2013a, b) and with recently engineered circadian clock mutants (Kotwica-Rolinska et al., 2019).

## Data Availability Statement

All datasets generated for this study are included in the article/Supplementary Material.

## Author Contributions

JK-R designed the study with input from DD and MK. MK, JK-R, and HV performed the experiments. MK analyzed the results. DD wrote the manuscript with input from all authors.

## Conflict of Interest

The authors declare that the research was conducted in the absence of any commercial or financial relationships that could be construed as a potential conflict of interest.
