# Supplementary figures and images for: Light and Temperature Synchronizes Locomotor Activity in the Linden Bug, Pyrrhocoris apterus
Source: Front Physiol. 2020 Apr 2;11:242. doi: 10.3389/fphys.2020.00242 (PMC7142227; doi:10.3389/fphys.2020.00242)

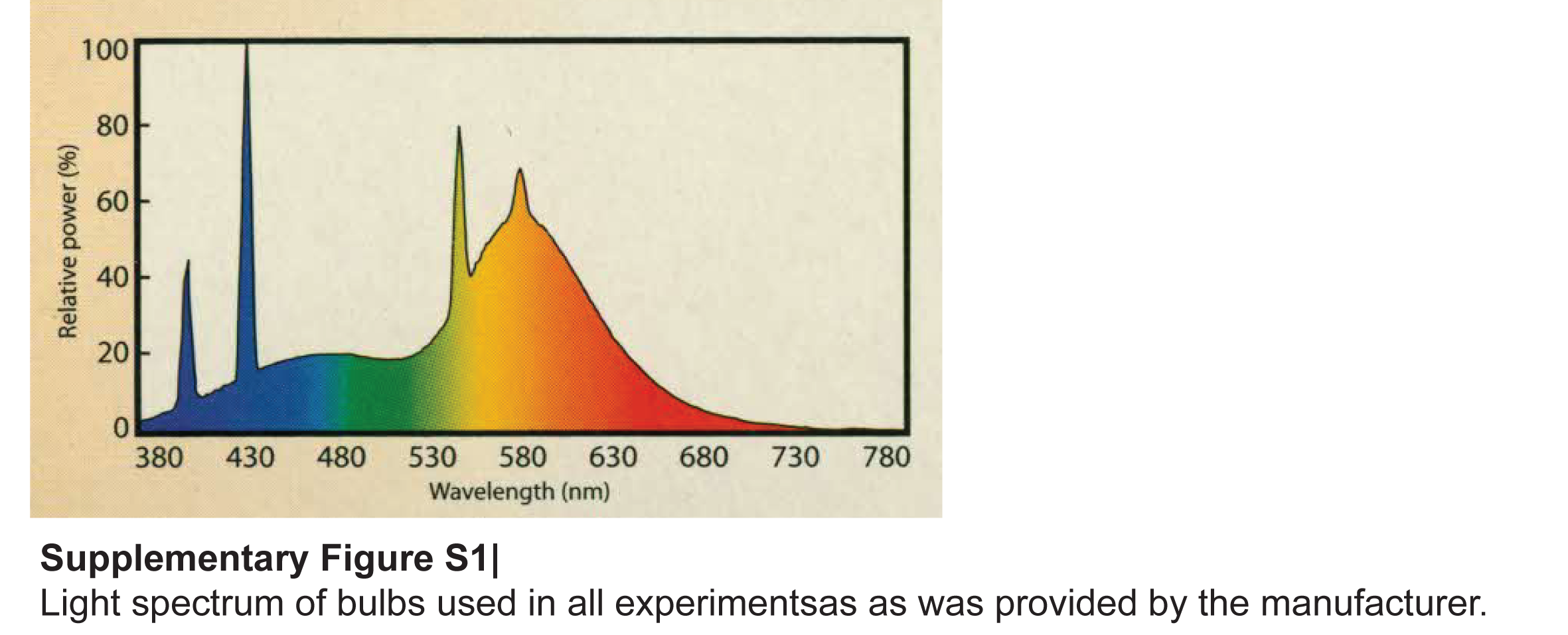

Supplement: Supplementary file 1 [file Image_1.tif]

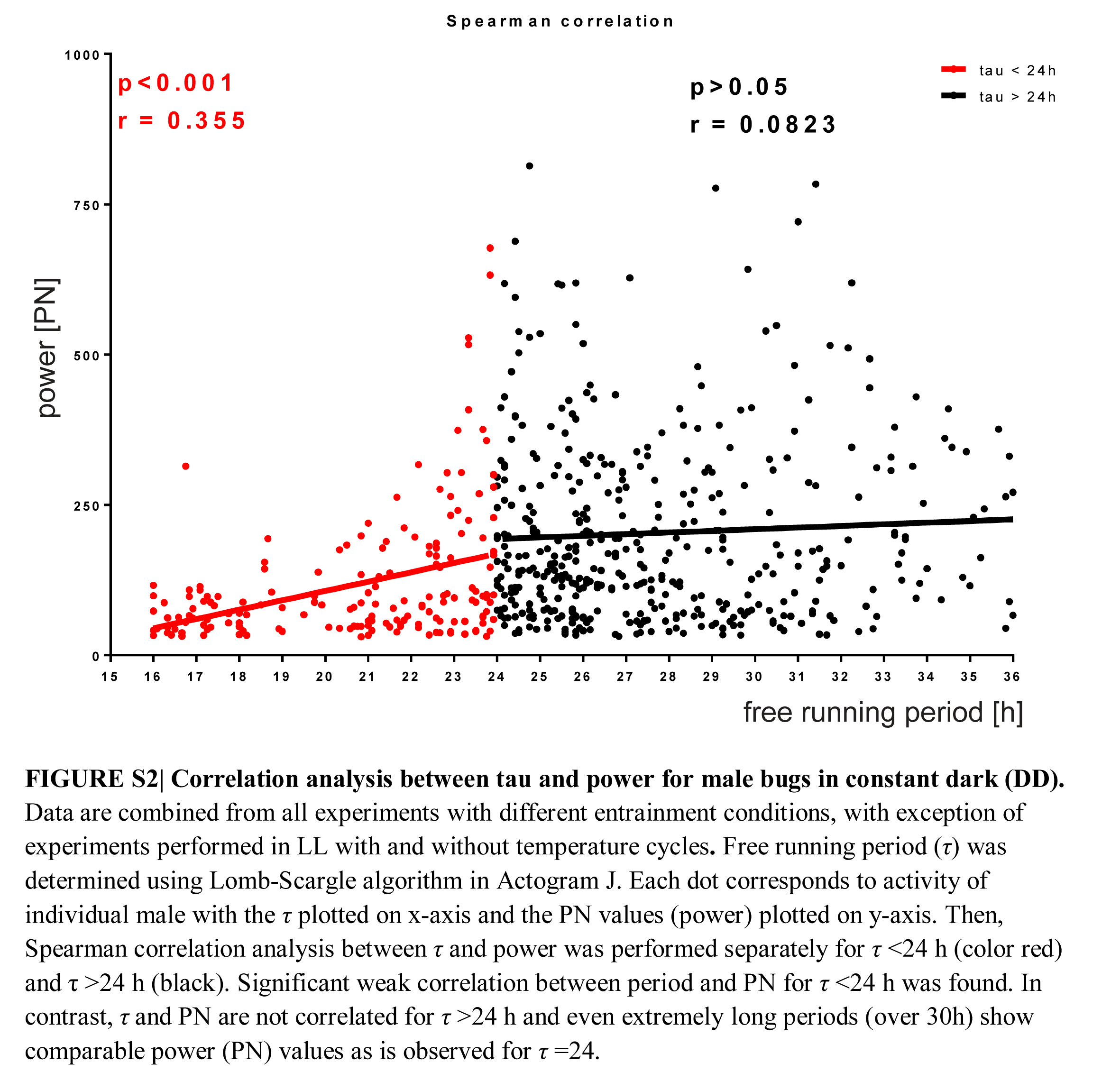

Supplement: Supplementary file 2 [file Image_2.TIF]

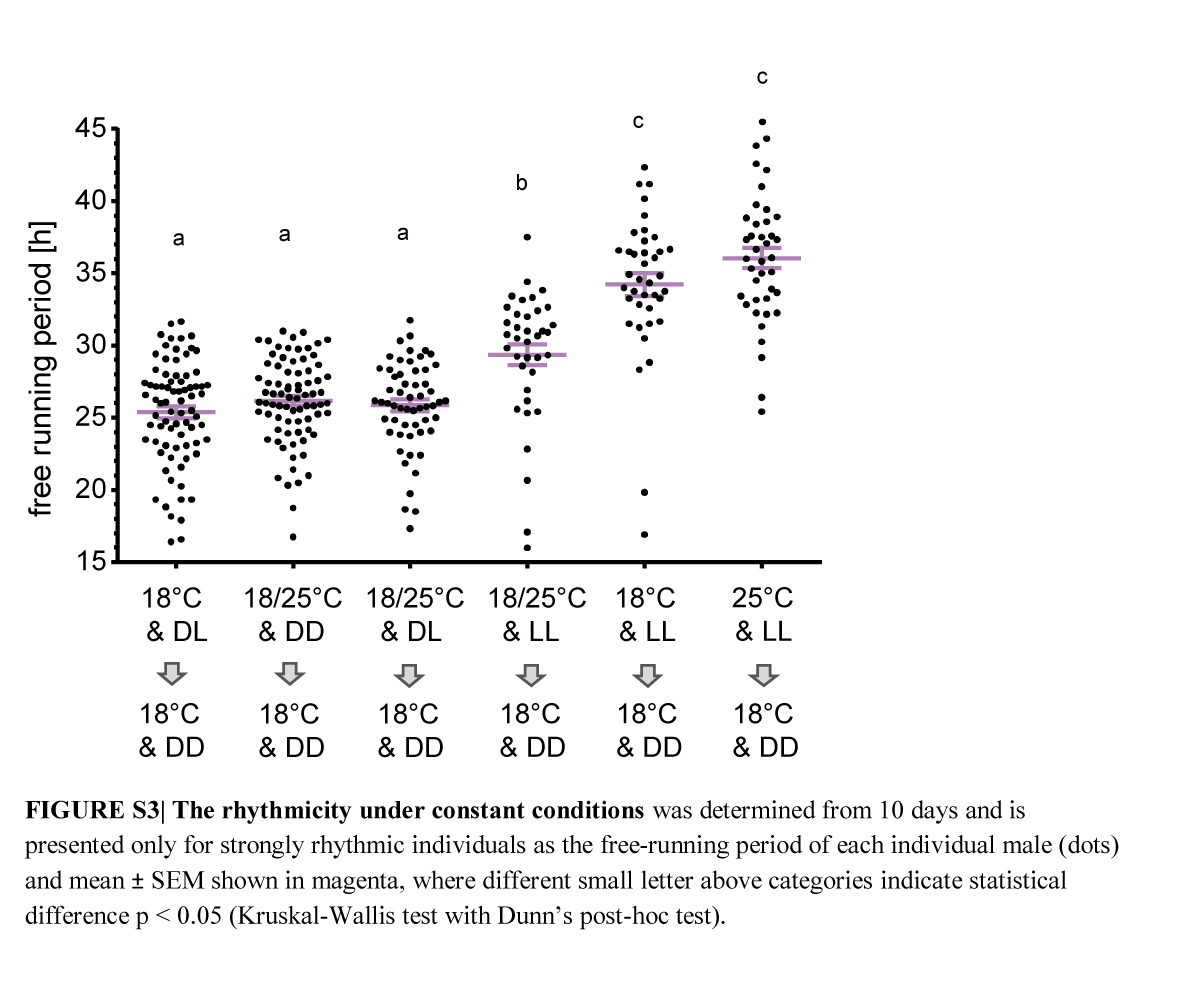

Supplement: Supplementary file 3 [file Image_3.TIF]
