# Supplementary material for: Light and Temperature Synchronizes Locomotor Activity in the Linden Bug, Pyrrhocoris apterus
Source: Front Physiol. 2020 Apr 2;11:242. doi: 10.3389/fphys.2020.00242 (PMC7142227; doi:10.3389/fphys.2020.00242)
Supplement: Supplementary file 4 [file Table_1.docx]

|  | |  |  |  | strongly rhythmic [%] | weakly rhythmic [%] | arrhythmic [%] | all bugs in experiment [n] | strongly rhythmic bugs [n] | weakly rhythmic bugs [n] | arrhythmic bugs [n] | TAU (mean from all strongly+weakly rhythmic bugs [h] | SEM (from all strong;y +weakly rhythmic bugs [h] |
| --- | --- | --- | --- | --- | --- | --- | --- | --- | --- | --- | --- | --- | --- |
|  | FIGURE 1 | |  |  |  |  |  |  |  |  |  |  |  |
|  | 25°C & DL -> 25°C DD | | | | 66.67 | 26.98 | 6.35 | 63 | 42 | 17 | 4 | 24.91 | 0.2116 |
|  | 18°C & DL -> 18°C & DD | | | | 80.41 | 16.5 | 3.09 | 97 | 78 | 16 | 3 | 25.07 | 0.4025 |
|  | 18/25°C & DD -> 18°C & DD | | | | 70.91 | 26.36 | 2.73 | 110 | 78 | 29 | 3 | 25.64 | 0.3412 |
|  | 18/25°C & DL -> 18°C & DD | | | | 89.06 | 10.94 | 0 | 64 | 57 | 7 | 0 | 25.92 | 0.4082 |
|  |  | |  |  |  |  |  |  |  |  |  |  |  |
|  | FIGURE 2 | |  |  |  |  |  |  |  |  |  |  |  |
|  | 25°C & DL -> 25°C & DD | | | | 86.6 | 6.7 | 6.7 | 30 | 26 | 2 | 2 | 25.18 | 0.1959 |
|  | 18/25°C & DD -> 18°C & DD | | | | 56.6 | 36.7 | 6.7 | 30 | 17 | 11 | 2 | 24.78 | 0.7636 |
|  | 18/25°C & LD -> 18°C & DD | | | | 73.3 | 23.3 | 3.4 | 90 | 66 | 21 | 3 | 24.43 | 0.3824 |
|  |  | |  |  |  |  |  |  |  |  |  |  |  |
|  | FIGURE 3 | |  |  |  |  |  |  |  |  |  |  |  |
|  | 18/25°C & DD -> 21.5°C & DD | | | | 84.9 | 11.32 | 3.78 | 53 | 45 | 6 | 2 | 26.13 | 0.2754 |
|  | 25/18°C & DD -> 21.5°C & DD | | | | 82.35 | 15.69 | 1.96 | 51 | 42 | 8 | 1 | 26.99 | 0.2807 |
|  |  | |  |  |  |  |  |  |  |  |  |  |  |
|  | FIGURE 5 | |  |  |  |  |  |  |  |  |  |  |  |
|  | 18°C & DL -> 18°C & DD | | | | 80.41 | 16.5 | 3.09 | 97 | 78 | 16 | 3 | 25.07 | 0.4025 |
|  | 18/25°C & DD -> 18°C & DD | | | | 70.91 | 26.36 | 2.73 | 110 | 78 | 29 | 3 | 25.64 | 0.3412 |
|  | 18/25°C & DL -> 18°C & DD | | | | 89.06 | 10.94 | 0 | 64 | 57 | 7 | 0 | 25.92 | 0.4082 |
|  | 18/25°C & LL -> 18°C & DD | | | | 69.81 | 26.42 | 3.77 | 53 | 37 | 14 | 2 | 27.31 | 0.8479 |
|  | 18°C & LL -> 18°C & DD | | | | 82.98 | 12.77 | 4.25 | 47 | 39 | 6 | 2 | 33.45 | 0.9131 |
|  | 25°C & LL -> 18°C & DD | | | | 81.63 | 18.37 | 0 | 49 | 40 | 9 | 0 | 35.76 | 0.686 |
|  |  | |  |  |  |  |  |  |  |  |  |  |  |
|  | FIGURE 6 | |  |  |  |  |  |  |  |  |  |  |  |
|  | 25°C & DL -> 25°C & DD | | | | 66.67 | 26.98 | 6.35 | 63 | 42 | 17 | 4 | 24.76 | 0.2513 |
|  | 25°C & DL -> 25°C & LL | | | | 41.07 | 41.07 | 17.86 | 112 | 46 | 46 | 20 | 24.01 | 0.4178 |
|  | 18/25°C & DD -> 18°C & DD | | | | 70.91 | 26.36 | 2.73 | 110 | 78 | 29 | 3 | 25.64 | 0.3412 |
|  | 18/25°C & DD -> 25°C & DD | | | | 41.758 | 31.868 | 26.374 | 91 | 38 | 29 | 24 | 24.31 | 0.3155 |
